# Supplementary material for: Microbial Populations in Ruminal Liquid Samples from Young Beefmaster Bulls at Both Extremes of RFI Values
Source: Microorganisms. 2023 Mar 6;11(3):663. doi: 10.3390/microorganisms11030663 (PMC10055678; doi:10.3390/microorganisms11030663)
Supplement: Supplementary file 1 [file microorganisms-11-00663-s001.zip › microorganisms-2197273-supplementary.pdf]

microorganisms

MDPI

Supplementary Information

Microbial Populations in Ruminal Liquid Samples from Beefmaster Steers at Both Extremes of RFI Values

Nelson Manzanares-Miranda [1,†], Jose F. Garcia-Mazcorro [2,†], Eduardo B. Pérez-Medina [2], Anakaren Vaquera-Vázquez [3], Alejandro Martínez-Ruiz [3], Yareellys Ramos-Zayas [2,4] and Jorge R. Kawas [4,5,*]

[1] Centro de Investigación en Producción Agropecuaria (CIPA), Universidad Autónoma de Nuevo León (UANL), Linares 67700, Mexico
[2] Research and Development, MNA de Mexico, San Nicolas de los Garza 66477, Mexico
[3] Laboratorio de Biología Molecular, Laboratorio Central Regional del Norte (LCRN), Guadalupe 67100, Mexico
[4] Posgrado Conjunto Agronomía-Veterinaria, UANL, General Escobedo 66054, Mexico
[5] Facultad de Agronomía, UANL, General Escobedo 66054, Mexico
* Correspondence: jorge.kawasgr@uanl.edu.mx
† These authors contributed equally to this work.

Citation: Manzanares-Miranda, N.; Garcia-Mazcorro, J.F.; Pérez-Medina, E.B.; Vaquera-Vázquez, A.; Martínez-Ruiz, A.; Ramos-Zayas, Y.; Kawas, J.R. Microbial Populations in Ruminal Liquid Samples from Beefmaster Steers at Both Extremes of RFI Values. *Microorganisms* **2023**, *11*, 663. https://doi.org/10.3390/microorganisms11030663

Academic Editor: Todd Riley Callaway

Received: 17 January 2023
Revised: 14 February 2023
Accepted: 1 March 2023
Published: 6 March 2023

Copyright: © 2023 by the authors. Licensee MDPI, Basel, Switzerland. This article is an open access article distributed under the terms and conditions of the Creative Commons Attribution (CC BY) license (https://creativecommons.org/licenses/by/4.0/).

1. Centered log-ratio (clr) transformation

As mentioned in the main text, the relative proportions of 16S reads have historically been the data of choice to perform comparisons of microbial taxa in studies of gut microbiota in ruminants and other animal species. However, it is well known that relative abundance can lead to spurious correlations, originally pointed out by Pearson more than a century ago (Pearson, 1896). The issue with the analysis of relative abundance has been discussed by contemporary researchers (Holmes et al. 2012; Fernandes et al. 2014; Mandal et al. 2015; Gloor and Reid 2016; Morton et al. 2019), and many methods and approaches have been implemented to deal with this issue (Nearing et al. 2022). Here, we focus on the centered log-ratio (clr) transformation (Moossavi et al. 2019).

There are at least two packages in R that can perform centered log-ratio (clr) transformation, the rgr and the compositions packages. Note that, unlike ANCOM (Mandal et al. 2015), available in the composition plugin in QIIME2 (https://docs.qiime2.org/2022.2/plugins/available/composition/), clr transformation in the rgr package is performed on the raw data not on the relative abundance (https://search.r-project.org/CRAN/refmans/rgr/html/clr.html). We think this is more appropriate because, as we showed above, the relative abundances contain data that do not necessarily reflect the nature of the original data.

Our analyses using both the rgr and the compositions packages in R showed that these transformations produce the same results when analyzing a full data set or fractions of the data set. In other words, the clr transformations in these packages seem not to take into consideration the number of samples. Also, these methods do not accept samples that contain any 0, which are common in 16S sequencing analyses that may or may not reflect true absence of the taxon in the environment (even a total of 500,000 16S sequences from a sample can fall short from 100 mg of intestinal contents containing ~1×10^{11} microbes per gram, Sender et al. 2016). On the other hand, our analysis showed that the formula for clr transformation [clr<-apply(log2(data+0.5), 2, function(x) x-mean(x))], explained in a tutorial from the QIIME2 forum (Bisanz 2018), produce different results depending on the number of samples. We think this is important because some researchers may want to explore the analysis of subsets of their data sets, for various reasons. It is important to point out that there is a perfect relationship between the sequence counts and the corresponding clr transformed data for each taxon when using the formula above, but not with the clr transformation provided by the R packages.

2. Diet used during feed efficiency test

Table S1. Diet used during feed efficiency test.

Ingredient	Composition (g/kg)
Ground corn	500
Klein grass hay	160
Distillers dried grains	100
Cane molasses [1]	80
Soybean meal	75
Whole cottonseed	60
Calcium carbonate	13.2
Urea	5.3
Salt	4
Mineral-vitamin premix [2]	2.5

[1] Cane molasses:water mix (50:50). [2] Trace minerals (Mn, 17,000 mg/kg; Zn, 34,000 mg/kg; Cu, 3,400 mg/kg; I, 170 mg/kg; Co, 68 mg/kg; Se, 102 mg/kg); vitamin A (880,000 I.U./kg), vitamin E (20,000 I.U./kg), and sodium monensin (12 mg/kg).

3. Variation analysis

We calculated the variation between time points, DNA extraction methods, and RFI groups, using both the relative abundance of taxa and the clr-transformed data at the phylum level. With the exception of Tenericutes, this analysis showed that the variation in microbial abundance was always higher between DNA extraction methods compared to the variation between days of sampling and between high (LRFI) and low (HRFI) efficiency animals.

Table S2. Standard deviation values obtained from two average values corresponding to 2 time points, 2 DNA extraction methods, and 2 RFI groups [1].

Taxon	Time points	DNA extraction methods	RFI groups
Actinobacteria	0.21	0.64	0.01
Actinobacteria_clr	0.34	1.33	0.35
Bacteroidetes	0.45	5.6	3.6
Bacteroidetes_clr	0.06	0.29	0.03
Chloroflexi	0.03	0.10	0.07
Chloroflexi_clr	0.28	1.37	1.19
Cyanobacteria	0.22	0.86	0.26
Cyanobacteria_clr	0.21	1.74	0.37
Elusimicrobia	0.01	0.25	0.12
Elusimicrobia_clr	0.25	2.28	1.03
Euryarchaeota	0.19	0.76	0.35
Euryarchaeota_clr	0.36	1.28	0.32
Fibrobacteres	0.06	0.63	0.07
Fibrobacteres_clr	0.45	3.61	0.04
Firmicutes	0.13	8.4	2.9
Firmicutes_clr	0.06	0.44	0.11
Lentisphaerae	0.04	0.07	0.02
Lentisphaerae_clr	0.36	0.84	0.09
Planctomycetes	0.18	0.65	0.16
Planctomycetes_clr	0.25	1.08	0.11
Proteobacteria	0.35	1.5	0.89

Proteobacteria_clr	0.35	0.89	0.21
Spirochaetes	0.16	2.5	0.10
Spirochaetes_clr	0.23	1.95	0.52
SR1	0.02	0.20	0.13
SR1_clr	0.21	2.22	0.88
Synergistetes	0.03	0.08	0.03
Synergistetes_clr	0.05	0.56	0.32
Tenericutes	0.22	0.07	0.20
Tenericutes_clr	0.38	0.09	0.38
TM7	0.28	1.4	0.86
TM7_clr	0.12	0.51	0.67
Unassigned phylum	0.08	1.2	0.29
Unassigned phylum clr	0.05	0.76	0.18
Verrucomicrobia	0.27	0.60	0.27
Verrucomicrobia_clr	0.14	0.18	0.14

1 For each taxon, we calculated the average relative abundance using all measurements at each time point, DNA extraction method, and RFI groups (20 measurements were used for each subgroup). The standard deviations in this Table were calculated using those two average values. The same procedure was performed using clr-transformed data. The only taxon that did not show higher standard deviation from the two DNA extraction methods using both the relative abundance and the clr-transformed data was Tenericutes (highlighted in gray).

4. Differences in microbial abundances between LRFI and HRFI

The raw number of 16S sequences were transformed using the formula [clr<-apply(log2(data+0.5), 2, function(x) x-mean(x))] as explained above, and the clr-transformed data was used to perform statistical comparisons. Table S2 to S4 show a summary of statistical results at the class, order, and genus level, respectively.

Table S3. Summary of statistical results (*p*-values) at the class level.[1.]

Class	Time points	DNA extraction methods	RFI groups
Actinobacteria (unassigned class)	0.2699	0.0075	0.2068
Alphaproteobacteria	0.8632	0.6694	0.1045
Anaerolineae	0.4963	0.0020	0.0059
Bacilli	0.2828	0.6502	0.0483
Bacteria (unassigned phylum)	0.8166	0.0012	0.4094
Bacteroidia	0.7190	0.1642	0.8513
Bacteroidetes (unassigned class)	0.9871	0.1591	0.5163
Betaproteobacteria	0.4443	<0.0001	0.6722
Chloroplast[2]	NA	NA	NA
Clostridia	0.7734	0.0005	0.5373
Coriobacteriia	0.1762	<0.0001	0.2174
Deltaproteobacteria	0.4492	0.3528	0.4589
Elusimicrobia	0.5559	<0.0001	0.3710
Endomicrobia[2]	NA	NA	NA
Epsilonproteobacteria	0.9730	<0.0001	0.4977
Erysipelotrichi	0.5833	0.0319	0.5407
Fibrobacteria	0.3888	<0.0001	0.9421
Firmicutes (unassigned class)	0.7646	<0.0001	0.3460
Flavobacteriia[3]	0.9556	<0.0001	0.6111

	NP (p = NS)	NP (<0.001)	NP (p = NS)
Gammaproteobacteria	0.2997	0.0001	0.2498
Lentisphaeria	0.4119	0.0579	0.8404
Methanobacteria	0.2126	<0.0001	0.2421
Mollicutes	0.1650	0.7420	0.3420
OD1 (unassigned class)[2]	NA	NA	NA
Opitutae[2]	NA	NA	NA
Planctomycetes[2]	NA	NA	NA
Planctomycetia	0.2728	<0.0001	0.6461
Proteobacteria (unassigned class)	0.6639	0.2088	0.7896
Spirochaetes	0.4740	<0.0001	0.1146
SR1 (unassigned class)	0.6005	<0.0001	0.0338
Synergistia	0.8530	0.0369	0.2161
Thermoplasmata[2]	NA	NA	NA
TM7-3	0.7447	0.1730	0.0731
Unassigned[2]	NA	NA	NA
Verruco-5	0.4920	0.5161	0.6316
4C0d2 (Cyanobacteria)	0.6343	<0.0001	0.4225

[1] We used PROC MIXED in SAS University Edition using the clr-transformed data from each taxon as dependent variable, and Day of Sampling, DNA extraction method, and RFI, as independent variables, without random effects. [2] Statistical analysis not performed because of the presence of 20 or more samples (>50%) with the same value, resulting from clr transformation of 0's. 3Residuals not normally distributed. NP: non-parametric analysis, NS: non-significant ($p > 0.05$).

Table S4. Summary of statistical results (p-values) at the order level.[1.]

**Order**	**Time points**	**DNA extraction methods**	**RFI groups**
Actinomycetales	0.2869	0.0108	0.2142
Aeromonadales	0.3896	0.0001	0.4258
Alphaproteobacteria	0.5496	0.0021	0.7941
Anaerolineales	0.4963	0.0020	0.0059
Bacteria (unassigned order)	0.8166	0.0012	0.4094
Bacteroidales[2]	0.7190	0.1642	0.8513
Bacteroidetes (unassigned order)	0.9871	0.1591	0.5163
Campylobacterales[2]	0.9730<br>NP (p = NS)	<0.0001<br>NP (<0.0001)	0.4977<br>NP (p = NS)
Clostridiales	0.7716	0.0005	0.5366
Coriobacteriales	0.1762	<0.0001	0.2174
CW040 (TM7)	0.7447	0.1730	0.0731
Desulfovibrionales	0.7143	0.6129	0.8524
Elusimicrobiales	0.5559	<0.0001	0.3710
Erysipelotrichales	0.5833	0.0319	0.5407
Fibrobacterales	0.3888	<0.0001	0.9421
Firmicutes (unassigned order)	0.7646	<0.0001	0.3460
Flavobacteriales[2]	0.9556<br>NP (p = NS)	<0.0001<br>NP (<0.0001)	0.6111<br>NP (p = NS)
Gammaproteobacteria	0.8686	0.0414	0.4152
Lactobacillales	0.1600	0.4156	0.0422

Methanobacteriales	0.2126	<0.0001	0.2421
Mycoplasmatales	0.8647	0.1578	0.3185
Pirellulales	0.2676	<0.0001	0.5400
Planctomycetia[2]	0.3929<br>NP (p = NS)	0.4248<br>NP (p = NS)	0.2589<br>NP (p = NS)
Proteobacteria (unassigned order)[2]	0.6639<br>NP (p = NS)	0.2088<br>NP (p = NS)	0.7896<br>NP (p = NS)
SR1	0.6005	<0.0001	0.0338
RF32	0.8552	<0.0001	0.0069
RF39	0.1437	0.3596	0.2903
Rhizobiales	0.6572	<0.0001	0.9995
Rhodospirillales	0.1754	0.2827	0.0160
Spirochaetales	0.4987	<0.0001	0.1383
Synergistales	0.8530	0.0369	0.2161
Victivallales[2]	0.9230<br>NP (p = NS)	0.0005<br>NP (<0.005)	0.6017<br>NP (p = NS)
WCHB141	0.4920	0.5161	0.6316
YS2	0.6343	<0.0001	0.4225
Z20	0.5161	0.0183	0.8541

[1] We used PROC MIXED in SAS University Edition using the clr-transformed data from each taxon as dependent variable, and Day of Sampling, DNA extraction method, and RFI, as independent variables, without random effects. [2] Residuals not normally distributed. A total of 21 taxa were not analyzed because of the presence of 20 or more samples (≥50%) with the same value, resulting from clr transformation of 0's (not shown in this table).

Table S5. Summary of statistical results (p-values) at the genus level organized by phylum.[1.]

Genus (or other level in case of unassigned genus)	Time points	DNA extraction method	RFI groups
**Actinobacteria**			
Brooklawnia	0.2999	0.0209	0.2865
Family Coriobacteriaceae (unassigned genus)[2]	0.2026	<0.0001	0.8036
Family Coriobacteriaceae (unassigned genus)[2]	0.5589	<0.0001	0.4996
Family Propionibacteriaceae (unassigned genus)[2]	0.7009	0.1138	0.1665
Olsenella	0.2181	0.0018	0.5642
**Bacteroidetes**			
Aureimonas	0.5471	<0.0001	0.3176
Bacteroidetes (unassigned genus)[2]	0.9871	0.1591	0.5163
BS11 (Bacteroidetes)	0.7732	0.0003	0.0021
CF231 (Bacteroidetes)	0.6653	0.0007	0.1996
Family Flavobacteriaceae (unassigned genus)[2]	0.5065	<0.0001	0.6760
Family Paraprevotellaceae (unassigned genus)	0.6600	0.0096	0.0567
Family Paraprevotellaceae (unassigned genus)	0.5164	0.7275	0.6610
Family Prevotellaceae (unassigned genus)	0.9666	0.0090	0.1212
Family RF16 (unassigned genus)	0.8892	<0.0001	0.0904
Family S24-7 (unassigned genus, Bacteroidetes)	0.7296	0.2016	0.0511
Order Bacteroidales (unassigned genus)	0.7721	<0.0001	0.2146
Order Bacteroidales (unassigned genus)	0.6329	0.0001	0.0421
Prevotella[2]	0.9708	0.9711	0.6491
YRC22[2]	0.7434	0.7973	0.5283
**Chloroflexi**			

SHD-231 (family Anaerolinaceae)	0.4963	0.0020	0.0059
**Cyanobacteria**			
Order YS2 (unassigned genus)	0.6343	<0.0001	0.4225
**Elusimicrobia**			
Family Elusimicrobiaceae (unassigned genus)	0.5870	<0.0001	0.4208
**Euryarchaeota**			
Methanobrevibacter	0.2126	<0.0001	0.2421
**Fibrobacteres**			
Fibrobacter	0.4188	<0.0001	0.8986
**Firmicutes**			
Anaerorhabdus[2]	0.9285	0.6946	0.9258
Anaerovibrio	0.7994	0.7566	0.0213
Bulleidia	0.5985	0.0108	0.2382
Butyrivibrio	0.5755	0.4963	0.6620
Clostridium (f. Lachnospiraceae)	0.9259	0.9774	0.1275
Clostridium (f. Clostridiaceae)	0.1339	0.0362	0.3363
Clostridium (f. Ruminococcaceae)	0.5560	0.1007	0.4855
Dialister	0.3692	0.9214	0.0141
Family Christensenellaceae (unassigned genus, Firmicutes)	0.5493	0.3561	0.0186
Family Erysipelotrichaceae (unassigned genus)	0.4634	0.8032	0.4393
Family Lachnospiraceae (unassigned genus)	0.1673	<0.0001	0.1259
Family Mogibacteriaceae (unassigned genus)	0.5086	0.9704	0.1637
Firmicutes (unassigned genus)	0.7646	<0.0001	0.3460
Marvinbryantia[2]	0.3946	0.0001	0.2419
Moryella[2]	0.5078	<0.0001	0.4231
Order Clostridiales (unassigned genus)	0.2639	0.0001	0.1492
Order Clostridiales (unassigned genus)	0.8309	0.3056	0.1174
Oscillospira	0.2201	<0.0001	0.2272
p-75-a5 (family Erysipelotrichaceae)	0.4341	0.0007	0.2731
RFN20 (family Erysipelotrichaceae)[2]	0.4028	<0.0001	0.7231
Ruminococcaceae (unassigned genus)	0.4693	0.0019	0.0538
Ruminococcaceae (unassigned genus)	0.7113	0.3873	0.5305
Ruminococcus	0.5957	0.1314	0.8909
Schwartzia	0.3115	0.4044	0.3993
Selenomonas	0.5023	0.0294	0.6883
Streptococcus	0.1600	0.4156	0.0422
Succiniclasticum	0.8930	0.0993	0.0011
Veillonellaceae (unassigned genus)	0.9588	0.3575	0.4578
Veillonellaceae (unassigned genus)	0.5769	0.4875	0.1387
**Lentisphaerae**			
Family R4-45B (unassigned genus)	0.5161	0.0183	0.8541
Victivallaceae (unassigned genus)[2]	0.9230	0.0005	0.6017
**Planctomycetes**			
Class Planctomycetia (unassigned genus)	0.3929	0.4248	0.2589
Family Pirellulaceae (unassigned genus)	0.2549	<0.0001	0.6108
Planctomycete	0.6384	0.0089	0.1762
**Proteobacteria**			
**Alphaproteobacteria**			

Family Acetobacteraceae (unassigned genus)	0.1754	0.2827	0.0160
Class Alphaproteobacteria (unassigned genus)[2]	0.5496	0.0021	0.7941
Order RF32 (unassigned genus)	0.8552	<0.0001	0.0069
Rhizobiales (unassigned genus)[2]	0.6572	<0.0001	0.9995
**Deltaproteobacteria**			
Desulfovibrio	0.4860	0.7426	0.3638
**Epsilonproteobacteria**			
Campylobacter[2]	0.9730	<0.0001	0.4977
**Gammaproteobacteria**			
Gammaproteobacteria (unassigned genus)	0.8686	0.0414	0.4152
Order Aeromonadales (unassigned genus)	0.9503	0.0251	0.0532
Ruminobacter	0.9151	0.5301	0.0286
Succinivibrio	0.9471	0.0153	0.3308
Succinivibrionaceae (unassigned genus)	0.2491	0.0004	0.8298
Succinivibrionaceae (unassigned genus)	0.2491	0.0004	0.8298
Proteobacteria (unassigned genus)	0.6639	0.2088	0.7896
**Spirochaetes**			
Spirochaetaceae (unassigned genus)	0.4071	<0.0001	0.2200
Spirochaetaceae (unassigned genus)	0.4071	<0.0001	0.2200
Treponema	0.4464	<0.0001	0.1050
**SR1**			
SR1 (unassigned genus)	0.6005	<0.0001	0.0338
**Synergistetes**			
Pyramidobacter	0.6613	0.0570	0.3432
**Tenericutes**			
Family Mycoplasmataceae (unassigned genus)[2]	0.8647	0.1578	0.3185
Order RF39 (unassigned genus)	0.1437	0.3596	0.2903
**TM7**			
Family F16 (unassigned genus)	0.7447	0.1730	0.0731
**Verrucomicrobia**			
Family RFP12 (unassigned genus)	0.4834	0.6394	0.7809
WCHB1-25 (unassigned genus)	0.6512	0.0054	0.2855
Order WCHB1-41 (unassigned genus)	0.5013	0.0006	0.4935
Order WCHB1-41 (unassigned genus)[2]	0.9639	0.0004	0.5196

[1] We used PROC MIXED in SAS University Edition using the clr-transformed data from each taxon as dependent variable, and Day of Sampling, DNA extraction method, and RFI, as independent variables, without random effects. [2] Residuals not normally distributed. The few taxa that show statistically significant difference between RFI groups are highlighted in gray.

References

1. Bisanz J. 2018. Tutorial: Integrating QIIME2 and R for data visualization and analysis using qiime2R (March 2020 Update v.0.99.20). https://forum.qiime2.org/t/tutorial-integrating-qiime2-and-r-for-data-visualization-and-analysis-using-qiime2r/4121 (accessed on May 2022).
2. Fernandes, A.D.; Reid, J.N.S.; Macklaim, J.M.; McMurrough, T.A.; Edgell, D.R.; Gloor, G.B. Unifying the analysis of high-throughput sequencing datasets: characterizing RNA-seq, 16S rRNA gene sequencing and selective growth experiments by compositional analysis. Microbiome 2014, 2, 15.
3. Gloor, G.B.; Reid, G. Compositional analysis: a valid approach to analyze microbiome high-throughput sequencing data. Can. J. Microbiol. 2016, 62, 692-703.
4. Holmes, I.; Harris, K.; Quince, C. Dirichlet multinominal mixtures: generative models for microbial metagenomics. PLoS ONE 2012, 7(2), e30126.
5. Mandal, S.; Van Treuren, W.; White, R.A.; Eggesbø, M.; Knight, R.; Peddada, S.D. Analysis of composition of microbiomes: a novel method for studying microbial composition. Microb. Ecol. Health Dis. 2015, 26, 27663.

6. Moossavi, S.; Sepehri, S.; Robertson, B.; Bode, L.; Goruk, S.; Field, C.J.; Lix, L.M.; de Souza, R.J.; Becker, A.B.; Mandhane, P.J.; et al. Composition and variation of the human milk microbiota are influenced by maternal and early-life factors. Cell Host & Microbe 2019, 25, 324-335.
7. Morton, J.T.; Marotz, C.; Washburne, A.; Silverman, J.; Zaramela, L.S.; Edlund, A.; Zengler, K.; Knight, R. Establishing microbial composition measurement standards with reference frames. Nature Comm. 2019, 10, 2719.
8. Nearing, J.T.; Douglas, G.M.; Hayes, M.G.; MacDonald, J.; Desai, D.K.; Allward, N.; Jones, C.M.A.; Wright, R.J.; Dhanani, A.S.; Comeau, A.M.; Langille, M.G.I. Microbiome differential abundance methods produce different results across 38 datasets. Nature Comm. 2022, 13, 342.
9. Pearson, K. Mathematical contributions to the theory of evolution — on a form of spurious correlation which may arise when indices are used in the measurement of organs. Proc. R. Soc. Lond. 1896, 60, 489-498.
10. Sender, R.; Fuchs, S.; Milo, R. Revised estimates for the number of human and bacteria cells in the body. PLoS Biol 2016, 14(8), e1002533.

Disclaimer/Publisher's Note: The statements, opinions and data contained in all publications are solely those of the individual author(s) and contributor(s) and not of MDPI and/or the editor(s). MDPI and/or the editor(s) disclaim responsibility for any injury to people or property resulting from any ideas, methods, instructions or products referred to in the content.
